# The impact of controlling diseases of significant global importance on greenhouse gas emissions from livestock production

**DOI:** 10.1186/s42522-023-00089-y

**Published:** 2023-12-08

**Authors:** Judith L. Capper

**Affiliations:** Livestock Sustainability Consultancy, Harwell, Didcot, Oxfordshire UK

**Keywords:** Animal health, Cattle, Environmental impact, Food security, Sustainability, Vaccines

## Abstract

**Background:**

A considerable body of evidence has reported the beneficial effects of improving productivity on reducing environmental impacts from livestock production. However, despite the negative impacts of animal diseases on reproduction, growth and milk production, there is little information available upon the impacts of animal disease on greenhouse gas emissions (GHGe). This study aimed to partially address this knowledge gap by investigating the effects of globally important vaccine-preventable diseases on GHGe from various livestock systems, namely: intensive dairy, extensive beef, commercial swine and backyard poultry production.

**Methods:**

Simple deterministic models were developed within Microsoft Excel to quantify the impacts of livestock disease on productivity (defined as total milk and/or meat yield, MMY) adjusted for disease prevalence both at the population level (high or low), and at the herd or flock level. Disease-induced changes in MMY were applied to the GHGe per kg of milk or meat according to the consequent changes in livestock populations required to maintain milk or meat production. Diseases investigated comprised foot and mouth, brucellosis, anthrax, lumpy skin disease, classical swine fever, porcine reproductive and respiratory syndrome (PRRS), low and high pathogenicity avian influenza (LPAI and HPAI), avian infectious bronchitis and Newcastle disease.

**Results:**

All diseases investigated had multifactorial impacts on total MMY, yet diseases that increased mortality in breeding or growing livestock (e.g. anthrax, classical swine fever and HPAI) showed greater impacts on GHGe per unit of milk or meat produced than those that primarily affecting yields or reproduction (e.g. brucellosis or LPAI). Prevalence also had considerable effects on potential GHGe. For example, maintaining backyard poultry meat production from a 100,000 hen population with 70% prevalence of HPAI increased GHGe by 11,255 MT CO_2_eq compared to a 30% prevalence at 3475 MT CO_2_eq above the baseline (0% prevalence). Effective reduction of the prevalence of PRRS in swine from 60 to 10%, FMD in beef cattle from 45 to 5% prevalence, or AIB in poultry from 75 to 20% prevalence would reduce GHGe intensities (CO_2_eq/kg CW) by 22.5%, 9.11% and 11.3% respectively.

**Conclusions:**

Controlling livestock disease can reduce MMY losses at the farm level, which improves food security, reduces GHGe and enhances livestock system sustainability.

**Supplementary Information:**

The online version contains supplementary material available at 10.1186/s42522-023-00089-y.

## Background

As the global population increases to a predicted 9.7 billion people by 2050 [1], consumption of milk, meat and eggs is predicted to increase by 48.6% by 2050 [2]. Livestock producers worldwide are therefore faced with the considerable task of producing more livestock-derived foods (LDF), using fewer resources, while maintaining food safety, quality and affordability [3].

There is a considerable body of evidence linking improved livestock productivity (milk and/or meat yield, MMY) and consequent decreases in livestock numbers with reduced resource use and greenhouse gas (GHGe) per unit of food produced, and therefore improved environmental sustainability [4–7]. Livestock health is a key determinant of system MMY, with losses manifested as reduced yields; decreased liveweight gains and therefore greater amounts of time needed to reach a target weight, maturity or parturition; impaired fertility; premature culling/mortality; or condemned organs and carcasses [8]. Although the MMY impacts of different diseases vary widely, the short and long-term effects of sub-clinical and clinical disease have potentially significant economic and environmental consequences [9]. This is a particular issue in smallholder or backyard operations, which constitute 82% of farms in low-income countries [10] and are associated with greater GHGe per unit of LDF [11, 12]. Such farms often have reduced access to the veterinary care, resources, infrastructure and political visibility that have facilitated intensification in higher-income countries, and, yet could reduce their environmental impacts considerably as a consequence of improved livestock health [13].

Considerable media coverage is devoted to the environmental impact of livestock production, including climate change, water and air pollution, soil erosion and biodiversity. Given public and industry concerns regarding climate change, GHGe are a critical environmental issue, yet there has been relatively little discussion of the role of livestock health as a GHG mitigation measure, and food production stakeholders often lack the information needed to make informed environmental decisions about disease treatment, control or elimination. Moreover, given the wide variation in production systems across the globe, it is difficult to draw conclusions regarding the impact of a disease on an entire sector (e.g. global dairy or beef production), and data is often lacking, especially in extensive livestock systems. The results of this study are therefore not intended to definitively address this knowledge gap, but to represent a starting point for the discussion, which may be expanded upon in future, especially as more robust data becomes available.

The objective of this study was to use simple deterministic models to quantify the changes in GHGe associated with controlling vaccine-preventable diseases of significant global importance within different species and production systems.

## Methods

The impacts of controlling livestock diseases upon GHGe per unit of milk or meat were assessed using simple deterministic Microsoft Excel-based population models based on livestock nutrition, performance and herd or flock population parameters. This study compiled and used health and production data from publicly available databases and peer-reviewed papers, therefore approval was not required from an ethics committee.

A selection of livestock production systems across the globe were chosen for analysis and verified by comparison to related literature that mapped global livestock systems [14]. Systems chosen comprised intensive dairy, extensive beef, commercial swine and backyard poultry. Although considerable breed, resource, climate and market variation exists even in systems that are similar in intensity, a number of diseases are ubiquitous within global production systems. However, considerable data gaps existed regarding the prevalence of many diseases, particularly in extensive, smallholder, or backyard systems – paucity of prevalence data therefore being a legitimate criticism of the current study. Examining the impacts of notifiable diseases was expected to overcome this hurdle to a certain extent, as although the recorded incidence data was not accurate at the farm-level, it provided a sense of the disease’s global importance. The OIE-WAHIS database [15], which reports the number of cases of notifiable diseases across the globe was therefore used to identify livestock diseases that fulfilled the first three (and preferentially the fourth) of the following criteria:

1. Significant global impact in terms of the number of cases per year or per outbreak, and therefore the number (head) of livestock lost globally
2. Outbreaks occurring across more than one global region
3. Controllable by vaccination (although vaccines may not be commercially available within every region or system due to infrastructure or regional veterinary regulations)
4. Zoonotic, thereby conferring a potential One Health risk.

Diseases were ranked according to the number (head) of livestock lost globally as a result of disease occurrence within the affected species [16], with the top 10 diseases for each species shown in Table 1, and the diseases highlighted (given the aforementioned selection criteria) chosen for analysis. Although all diseases chosen fitted the criteria, they varied in terms of impact on productivity (total milk or meat yield), acuteness of symptom onset and organs or systems affected. Furthermore, the selected diseases included those caused by either bacteria (brucellosis, anthrax) or viruses (food and mouth disease (FMD); lumpy skin disease (LSD); classical swine fever (CSF); porcine reproductive and respiratory syndrome (PRRS); both low and high pathogenicity avian influenza (LPAI and HPAI); avian infectious bronchitis (AIB); and Newcastle disease).

**Table 1:** Top 10 global diseases reported by the World Organisation for Animal Health (OIE) for various livestock species, ranked by the number of livestock units lost annually^a,b^

Ranking	Cattle	Swine	Poultry
1	Echinococcosis	**Classical swine fever**^**b**^	**High pathogenicity avian influenza**
2	Bovine tuberculosis	Swine vesicular disease	**Avian infectious bronchitis**
3	Enzootic bovine leukosis	**Porcine reproductive and respiratory syndrome**	**Low pathogenicity avian influenza**
4	**Brucellosis**	African swine fever	**Newcastle disease**
5	Haemorrhagic septicaemia	Aujeszky's disease	Infectious bursal disease
6	**Foot and mouth disease**	Echinococcosis	Mycoplasmosis
7	Rabies	Porcine cysticercosis	Pullorum disease
8	**Anthrax**	Leptospirosis	Fowl cholera
9	**Lumpy skin disease**	Foot and mouth disease	Fowl typhoid
10	Theileriosis	Bovine tuberculosis	Marek’s disease

^a^[16]

^b^ Diseases in bold font are those chosen for analysis within this study, although data on the impacts of lumpy skin disease on dairy production are not included within this paper as this disease is considered to be of limited threat to cattle in intensive dairy systems

**Fig. 1:**
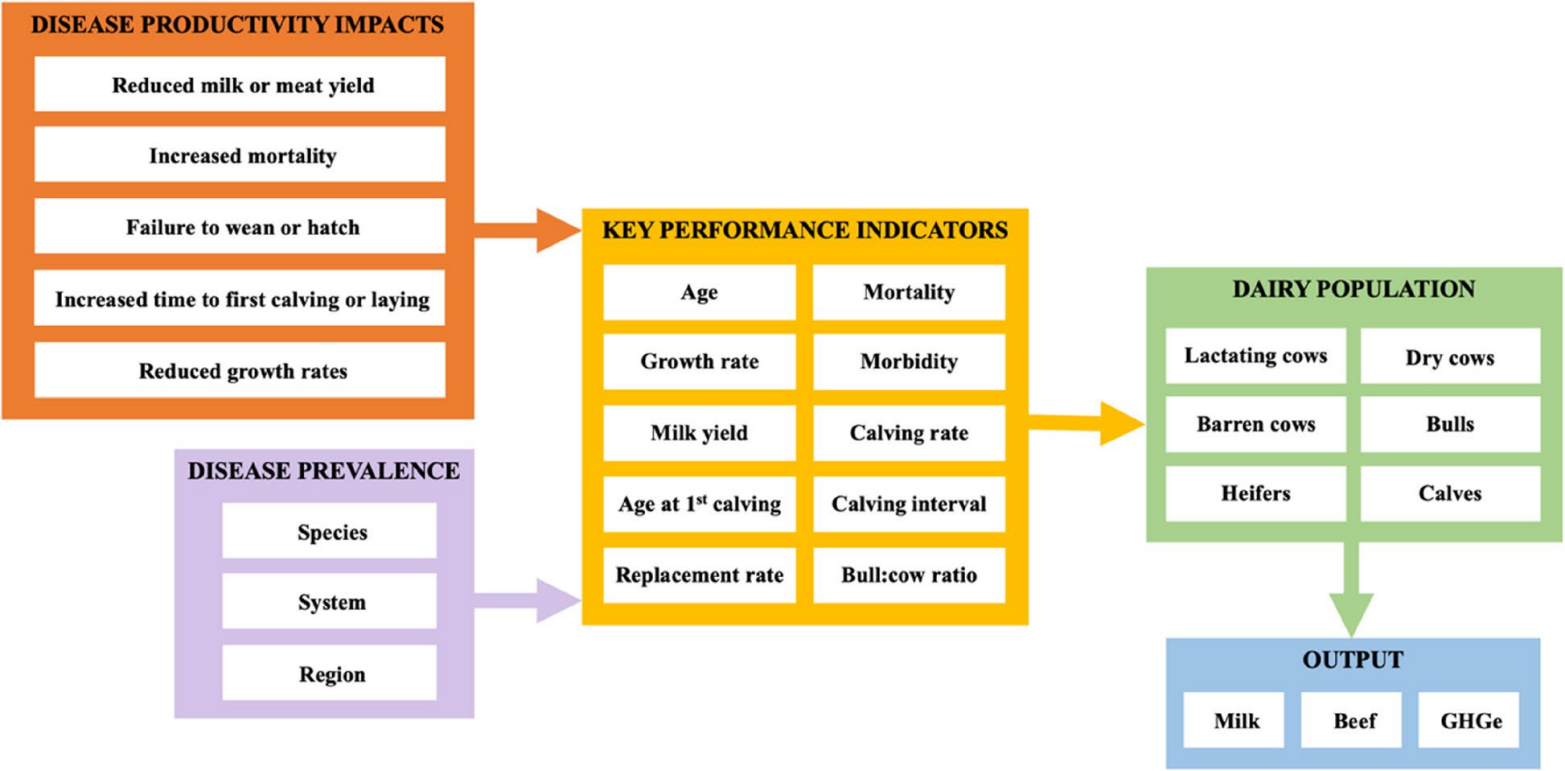
Simplified example of the dairy model used within the analysis

Simple deterministic livestock population models were developed within MS Excel to assess the impacts of disease on GHGe – an example relating to the dairy analysis is summarised in Fig. 1, with all models following a similar approach. Baseline herd or flocks (dependent on species) were modelled according to production parameters according to data shown in Tables 2, 3, 4, 5. The effect of specific diseases on GHGe were investigated based on the premise that, compared to the baseline level of performance for the specific region and system, disease would have a negative impact upon average herd or flock performance. A disease-challenged population would exhibit mortality- and morbidity-induced changes in MMY, with concurrent increases in the number of animals or time required to maintain LDF production and therefore the GHGe associated with producing a set quantity of milk or meat (Fig. 1).

**Table 2:** Baseline dairy cattle performance parameters

Performance parameter	Value
Annual milk yield (kg FPCM)^a,b^	4500
Age at first calving (mo)^c^	26.0
Calving interval (mo)^c^	14.0
Live calves born per cow per year^d^	0.81
Calves destined as heifer replacements^e^	0.29
Calves destined as beef cattle^f^	0.50
Proportion of lactating cows in the population^c^	0.45
Proportion of dry cows in the population^c^	0.06
Proportion of replacement heifers in the population^c^	0.48
Proportion of bulls in the population^c^	0.01
Proportion of replacement heifers born that enter herd^c^	0.85
Replacement rate (%)^c^	25.0
Calf and growing cattle mortality (%)^c^	5.0
Milk lost per cow death (kg FPCM)^b,g^	8357
Milk lost per non-yielding day of lactation (kg FPCM)^b,h^	14.4
GHGe per kg FPCM (kg CO_2_eq)^i^	1.50
Annual GHGe per dairy cow in the herd (kg CO_2_eq)^,j,k^	6750
Proportion of GHGe attributed to herd maintenance^c^	0.58
Proportion of GHGe attributed to lactation^c^	0.42

^a^[17]

^b^ FPCM, fat and protein corrected milk

^c^[5]

^d^ Calculated from calving rate, calving interval and calf mortality

^e^ Calculated from heifer replacement rate and 15% heifer mortality from birth to entering the herd

^f^ Calculated from total calves born, minus those required as replacements, adjusted for calf mortality

^g^ Calculated from annual milk yield, calving interval and age at first calving

^h^ Calculated from annual milk yield and lactation length

^i^[18]

^j^ Calculated from annual milk yield and GHGe per kg FPCM

^k^ Includes GHGe from all cattle in the herd, divided by the number of lactating cows

**Table 3:** Baseline beef cattle performance parameters

Performance parameter	Value
Age at first calving (mo)^a^	40.0
Calving interval (mo)^a^	16.0
Live calves born per cow per year^b^	0.53
Calves destined as heifer replacements^c^	0.14
Calves destined as beef cattle^d^	0.37
Age at weaning (mo)^a^	8.0
Finished cattle age at slaughter (mo)^a^	36.0
Finished cattle weight at slaughter (kg)^a^	530
Finished cattle carcass weight (kg)^a^	281
Proportion of cows in the population^a^	0.26
Proportion of replacement heifers in the population^a^	0.14
Proportion of bulls in the population^a^	0.04
Proportion of growing cattle in the population^a^	0.56
Proportion of replacement heifers born that enter herd^a^	0.90
Replacement rate (%)^a^	12.5
Calf and growing cattle mortality (%)^a^	5.0
Potential prime beef lost per cow death (kg CW)^e^	345
Cull cow beef lost per cow death (kg CW)^f^	214
GHGe per kg CW beef (kg CO_2_eq)^g^	73.0
Annual GHGe per beef cow in the herd (kg CO_2_eq)^h^	7997
Proportion of GHGe attributed to herd maintenance^a^	0.52
Proportion of GHGe attributed to cattle growth^a^	0.48

^a^[4]

^b^ Calculated from calving rate, calving interval and calf mortality

^c^ Calculated from heifer replacement rate and 10% heifer mortality from birth to entering the herd

^d^ Calculated from live calves born per year adjusted for post-birth mortality and heifers required as herd replacements

^e^ Calculated from live calves born per year. the calves that would otherwise have born in the time taken to rear a heifer to replace that cow (age at first calving) and the CW beef yield per animal

^f^ Calculated based on a 450 kg cow liveweight, 5% mortality rate and 50% dressing percentage

^g^[12]

^h^ Calculated based on GHGe from all cattle in the herd, divided by the number of cows and adjusted for GHGe associated with herd maintenance (vs. production) and calving interval

**Table 4:** Baseline swine performance parameters

Performance parameter	Value
Age at first farrowing (d)^a^	367
Annual litters per sow^b^	2.00
Annual pigs sold per sow (head)^c^	19.4
Finished pig age at slaughter (mo)^d^	8.40
Finished pig weight at slaughter (kg)^e^	116
Finished pig carcass weight (kg)^e^	83.6
Mean number of growing pigs in the herd per sow (head)^f^	13.6
Proportion of sows in the population^g^	0.07
Proportion of growing pigs in the population^h^	0.93
Potential growing pigs lost per sow death (head)^j^	19.4
GHGe per kg pigmeat (kg CO_2_eq)^j^	6.07
GHGe per kg pigmeat attributed to sows (kg CO_2_eq)^k^	1.15
GHGe per kg pigmeat attributed to growing pigs (kg CO_2_eq)^k^	4.92

^a^[19, 20]

^b^ Based on 2.35 litters per sow in intensive systems and 1.65 litters per sow in extensive systems

^c^[21–23]

^d^ Based on 170 d age at slaughter in intensive systems and 365 d age at slaughter in extensive systems

^e^[17]

^f^ Based on the age at slaughter and number of pigs produced per sow annually

^g^ The reciprocal of the mean number of growing pigs in the population per sow

^h^ 1 – the reciprocal of the mean number of growing pigs in the population per sow

^i^ Calculated from live pigs born per year. the pigs that would otherwise have been born in the time taken to rear a gilt to replace that sow (age at first farrowing) and the CW pigmeat yield per animal

^j^[11]

^k^ Calculated based on data from [24] applied to that of[11]

**Table 5:** Baseline poultry performance parameters

Performance parameter	Value
Mature hen bodyweight (kg)^a^	1.00
Age at maturity (mo)^a^	6.28
Age at culling (mo)^b^	55.2
Annual eggs produced per hen^a^	102
Proportion of eggs that are fertile^a^	0.62
Proportion of eggs that are hatchable^a^	0.64
Proportion of hatched chickens that survive to slaughter weight^a^	0.45
Annual total chickens per hen that survive to slaughter weight (birds)^c^	18.2
Chicken age at slaughter (d)	112
Chicken slaughter weight (kg)	0.90
Chicken carcass weight (kg)^d^	0.63
Mean number of growing chickens in the flock per hen (birds)^e^	5.51
Proportion of hen liveweight in the population^f^	0.29
Proportion of growing chicken liveweight in the population^g^	0.71
Potential growing chickens lost per hen death (birds)^h^	9.0
GHGe per kg chicken meat (kg CO_2_eq)^i^	6.60
GHGe per kg chicken meat attributed to hens (kg CO_2_eq)^j^	1.88
GHGe per kg chicken meat attributed to growing chickens (kg CO_2_eq)^j^	4.72

^a^[25]

^b^[26]

^c^[25, 27, 28]

^d^ Calculated at 70% dressing percentage

^e^ Calculated from the number of growing chickens surviving to slaughter and the average laying pattern across the year

^f^ Calculated from the number of growing chickens surviving to slaughter, the average weight and slaughter age of growing chickens, and hen liveweight

^g^ 1 – the proportion of hen liveweight in the population

^h^ Calculated from live chicks hatched per year. the chicks that would otherwise have been hatched in the time taken to rear a hen replacement (age at first laying) and the CW yield of poultrymeat per animal

^i^[11]

^j^ Calculated based on relative proportions of bird liveweight within the population

Within each model, impacts of disease on GHGe were calculated based on the prevalence of the disease, the MMY impacts, the duration of the disease and the livestock groups affected. The effects of disease in the challenged populations were sourced from the literature and are shown in Tables 6, 7. Diseases were assumed to occur in isolation, without effects of concurrent or secondary disease occurring via immunosuppression. Effects of changes in MMY conferred by livestock disease were quantified at the population level and normalised to a prevalence of 15% to compare the effects of disease across species. The effects of disease at low and high prevalence (derived from the literature and varying according to the disease) compared to the baseline (no disease) were then evaluated at the population and herd or flock level. Mortality was assumed to occur, on average, halfway through the year or production cycle, unless otherwise specified (e.g. stillbirth or abortion). Production losses were quantified in terms of total yield losses in milk and calves that could have been reared as beef animals (dairy); or carcass weight (CW) meat (beef, pork and chicken). A brief description of each model follows.

**Table 6:** Impacts of cattle diseases on performance parameters

Disease	Performance parameter	Impact in diseased animal
Foot and mouth disease	Milk yield	Acute drop (80%) and slow recovery over 105 days, 14.8% reduction over whole lactation (dairy cows) and 19.3% reduction over whole lactation (beef cows)^a^
	Reproduction	10% of cows abort^b^
	Heifer replacements	7.5% decrease in ADG, so age at first calving delayed to 28.1 mo (dairy heifers), and 19.3% decrease in ADG, so age at first calving delayed to 50.0 mo (beef heifers)
	Cow mortality	10% of infected cows culled^b^
	Heifer mortality	3% increase
	Bull mortality	3% increase
	Beef cattle ADG	19.3% decrease in pre-weaning ADG; 5% decrease post-weaning
Brucellosis	Milk yield	15.0% reduction over whole lactation (dairy and beef)^c^
	Reproduction	15% of cows abort, calving interval increased by 2 mo in cows that abort, 20% of aborting cows permanently sterile^c^
	Heifer replacements	Greater numbers required to replace cows that are sterile or die^c^
	Cow mortality	1% of cows that abort, die (0.15%)^c^
	Heifer mortality	10% mortality in calves from infected cows^c^
	Bull mortality	10% mortality in calves from infected cows^c^
	Beef cattle ADG	15.0% decrease in pre-weaning ADG; 5% decrease post-weaning
Anthrax	Milk yield	40.0% reduction over whole lactation as cows assumed to die, on average at 150 d into lactation (dairy and beef)
	Reproduction	N/A
	Heifer replacements	Greater numbers required to replace cows that are sterile or die
	Cow mortality	Dependent on ingestion of anthrax spores
	Heifer mortality	Dependent on ingestion of anthrax spores
	Bull mortality	Dependent on ingestion of anthrax spores
Lumpy skin disease	Milk yield	Acute drop (83.2%) for 70 d, 19.6% reduction over whole lactation (dairy cows) and 37.5% reduction over whole lactation (beef cows)^d^
	Reproduction	20% of cows abort, calving interval increased by 3 mo in 50% of cows, 10% of bulls culled because of orchiditis^e^
	Heifer replacements	Greater numbers required to replace cows that are sterile or die
	Cow mortality	Up to 5%, plus 5% culled due to chronic complications, 7% overall average^f^
	Heifer mortality	Up to 5%^f^
	Bull mortality	Up to 5%^f^
	Beef cattle ADG	5.9% decrease in pre-weaning ADG based on 32% of pre-weaning growth affected by milk yield decrease and milk accounting for 50% of intake

^a^[29]

^b^[30]

^c^[31]

^d^[32]

^e^[33, 34]

^f^[34, 35]

**Table 7:** Impacts of pig and poultry diseases on performance parameters

Disease	Performance parameter	Impact in diseased animal
**Pigs**		
Classical swine fever	Sow mortality	100% fatal^a^
	Reproduction	Zero due to mortality rate. 50% reduction overall assuming sows die halfway through the production cycle
	Pre-weaned pig mortality	100% fatal^a^
	Growing/finishing pig mortality	100% fatal^a^
	Growing/finishing pig ADG	N/A due to mortality rate
	Pig carcass weight	N/A due to mortality rate
Porcine reproductive and respiratory syndrome	Sow mortality	N/A
	Reproduction	19.3% of sows abort, 2.4% decrease in farrowings per sow^b^
	Pre-weaned pig mortality	75% of affected pigs die^c^
	Growing/finishing pig mortality	8.5% of affected pigs die^d^
	Growing/finishing pig ADG	25% decrease for the four weeks after weaning^e^
	Pig carcass weight	N/A
**Poultry**		
Low pathogenicity avian influenza	Hen mortality	Increased by 3.2%^f^
	Laying rate	Reduced by 74% for 14 d^f^
	Chick mortality	N/A
	Chicken ADG	Reduced by 3.2% for 14 d^g^
	Chicken carcass weight	N/A
High pathogenicity avian influenza	Hen mortality	100% fatal^h^
	Laying rate	Zero due to mortality rate. 50% reduction overall assuming birds die halfway through the production cycle
	Chick mortality	100% fatal^h^
	Chicken ADG	N/A due to mortality rate
	Chicken carcass weight	N/A due to mortality rate
Avian infectious bronchitis	Hen mortality	Increased by 5.0%^h^
	Laying rate	Reduced by 30% for up to 80 d^i^
	Chick mortality	Increased by 20%^j^
	Chicken ADG	Reduced by 30% for 12 d^i^
	Chicken carcass weight	N/A
Newcastle disease	Hen mortality	Increased by 15.0%^k^
	Laying rate	Reduced by 40% for up to 28 d^k^
	Chick mortality	Increased by 40%^k^
	Chicken ADG	Reduced by 50% for 10 dl
	Chicken carcass weight	N/A

^a^[15]

^b^[36, 37]

^c^[38]

^d^[37, 39]

^e^[37]

^f^[40]

^g^[41]

^h^[42]

^i^[43]

^j^[44, 45]

^k^[46]

^l^[47]

The dairy cattle model was founded upon intensive production systems characteristic of North America, Western Europe and Oceania, with dairy herds containing lactating and dry cows, replacement heifers and bulls at proportions according to those modelled in previous studies [5] and shown in Table 2. Although intensive systems predominate in the aforementioned regions where diseases such as FMD are seldom found, similar production systems are found worldwide, especially in developing dairy industries in Africa or Asia, and often in areas where FMD and other diseases in this study are endemic. Annual milk yields were set at 4,500 kg fat and protein corrected milk (FPCM) derived from the FAOSTAT database [17]. The MMY losses conferred by a cow dying were calculated according to the yields that would be produced over the time taken to rear a heifer to first calving (26 mo), considering a 14 mo calving interval, and were equal to 8,357 kg FPCM. A maximum of 14.4 kg of FPCM was lost per non-yielding day of lactation. The potential beef losses (calves that could otherwise be sold and reared for beef) incurred by cow mortality derived from the number of calves produced each year (0.81 per cow); a 5% calf mortality rate; the proportion of calves required as heifer replacements (0.29 per cow) accounting for a 25% culling rate and 85% of replacement heifers successfully entering the herd [5]. The effects of morbidity upon MMY were assessed based upon a 50,000 head population and a herd containing 200 lactating cows (plus associated dry cows, heifer replacements, bulls and growing/finishing cattle).

The GHGe for the baseline population was derived from a previous UN FAO study at 1.50 kg CO_2_eq per kg FPCM [18]. Fractions of GHGe were attributed to the resource requirements for lactation and maintenance for different cattle groups within the herd according to the previously reported proportions [5] at 42% for lactation and 58% for maintenance (25% lactating cows, 5% dry cows, 27% replacement heifers, 1.0% bulls). Dairy calves were assumed to be sold off-farm for beef soon after birth, therefore their GHGe were considered *de minimus*. After accounting for herd performance and population characteristics, the annual GHGe per lactating cow in the herd (including emissions from non-productive cattle) was calculated to be 6,750 kg CO_2_eq.

The effects of disease on extensive beef cattle production were modelled upon a baseline population characteristic of those found in South America, Northern Australia and South Africa, with cattle performance largely dictated by seasonal forage availability. The proportions of cows, bulls, replacement heifers and bulls, and growing cattle destined for beef were derived from a previous study [4], as shown in Table 3. Accounting for a 40 mo age at first calving and 16 mo calving interval, cows produced 0.53 live calves per year, weaned at 8.0 mo [4]. When adjusted for mortality, and a 12.5% replacement rate, 0.14 calves per cow were destined as heifer replacements, and 0.37 calves for beef. Cattle destined for beef were finished at 36 mo, yielding 280.9 kg CW. The MMY losses incurred by a beef cow dying were calculated according to the potential CW lost over the time required to rear an extra heifer replacement (344.7 kg CW) plus the cull cow beef lost by premature death (213.8 kg CW). Morbidity effects upon MMY were based upon a 50,000 head population and a herd containing 200 breeding cows (plus associated heifer replacements, bulls and growing/finishing cattle).

The GHGe for the baseline population were characteristic of extensive beef production at 73.0 kg CO_2_eq per kg CW [12]. As in the dairy model, the proportions of GHGe attributed to different cattle groups were proportional to their resource requirements as derived from a previous study at 52% for maintenance (38% cows, 10% replacement heifers, 5% bulls, 47% growing and finishing cattle) and 48% for growth [4]. The GHGe associated with the beef cattle population divided by the number of head of cows, produced annual GHGe per cow of 7,997 kg CO_2_eq.

Compared to ruminant systems, monogastric (swine and poultry) production across the globe have GHGe that are more dependent upon resource inputs (primarily feed) than cattle systems [11, 12]. Within the current study, the impacts of disease on swine production were assessed in commercial (non-backyard) operations, whereas effects in poultry systems were confined to backyard flocks. It should be noted that these are not and should not be assumed to be direct comparisons, but to provide a variety of systems within the study and to acknowledge the vital contribution made by backyard poultry to human nutrition and health worldwide.

Swine production was characterised by populations comprising sows, weaned pigs and growing/finishing pigs (Table 4), with sows first farrowing at 367 days of age, producing 2.00 litters per year, and selling 19.4 finished pigs per year [19–23]. Swine were finished at 8.4 mo of age and 116 kg liveweight, producing 83.6 kg CW pigmeat per head, the mean global commercial weight [17]. Given the age at slaughter and the annual number of pigs produced per sow, across the year there would be an average of 13.6 growing pigs per sow in the population at any time-point, with 6.9% of the swine population represented by sows and 93.1% by growing pigs. This analysis did not account for replacement gilts—if sows died, it was assumed that MMY declined because herd size decreased, rather than gilts being diverted from meat to replacements. Deaths of generic pigs (i.e. those that were neither specifically designated as sows or growing pigs) were associated with potential losses of a further 2.26 pigs. Disease effects were based upon a 100,000-sow population and a herd containing 1,000 breeding sows (plus associated growing pigs). Baseline swine population GHGe were derived from a UN FAO study [11] at 6.07 kg CO_2_eq per kg pigmeat CW, with fractions of GHGe attributed to sows (1.15 kg CO_2_eq) and growing pigs (4.92 kg CO_2_eq) within the herd [24].

Backyard poultry systems have no defined structure per se, and therefore may have myriad definitions. The impacts of morbidity and mortality on backyard poultry production within the current study were based upon a flock structure derived from published hen performance characteristics and shown in Table 5 [25–28]. Hens reached a mature bodyweight of 1.0 kg at 6.28 mo of age and were kept in the flock until culling at 55.2 mo. Accounting for the number of eggs laid (102 eggs), egg fertility (62%) and hatchability (64%) and the pre-slaughter mortality rate of growing chickens (55%) meant that each hen in the flock produced an average of 18.2 birds that survived to slaughter weight per year. Chickens were slaughtered at 112 d of age, at 0.90 kg liveweight (0.63 kg CW). At any time point 5.51 growing chickens would be present in the flock per hen, and the death of a generic bird (not specified as to whether it was a hen or a growing chicken) was associated with potential losses of 2.23 further birds. Impacts of poultry disease were based upon a 100,000-hen population plus a flock of 10 hens (plus associated growing chickens). The GHGe per kg poultry meat CW was 6.60 kg CO_2_eq with 1.88 kg CO_2_eq of the emissions attributed to hens and the remaining 4.72 kg CO_2_eq to growing chickens, based on relative proportions of bird liveweight within the population [11].

## Results

**Fig. 2:**
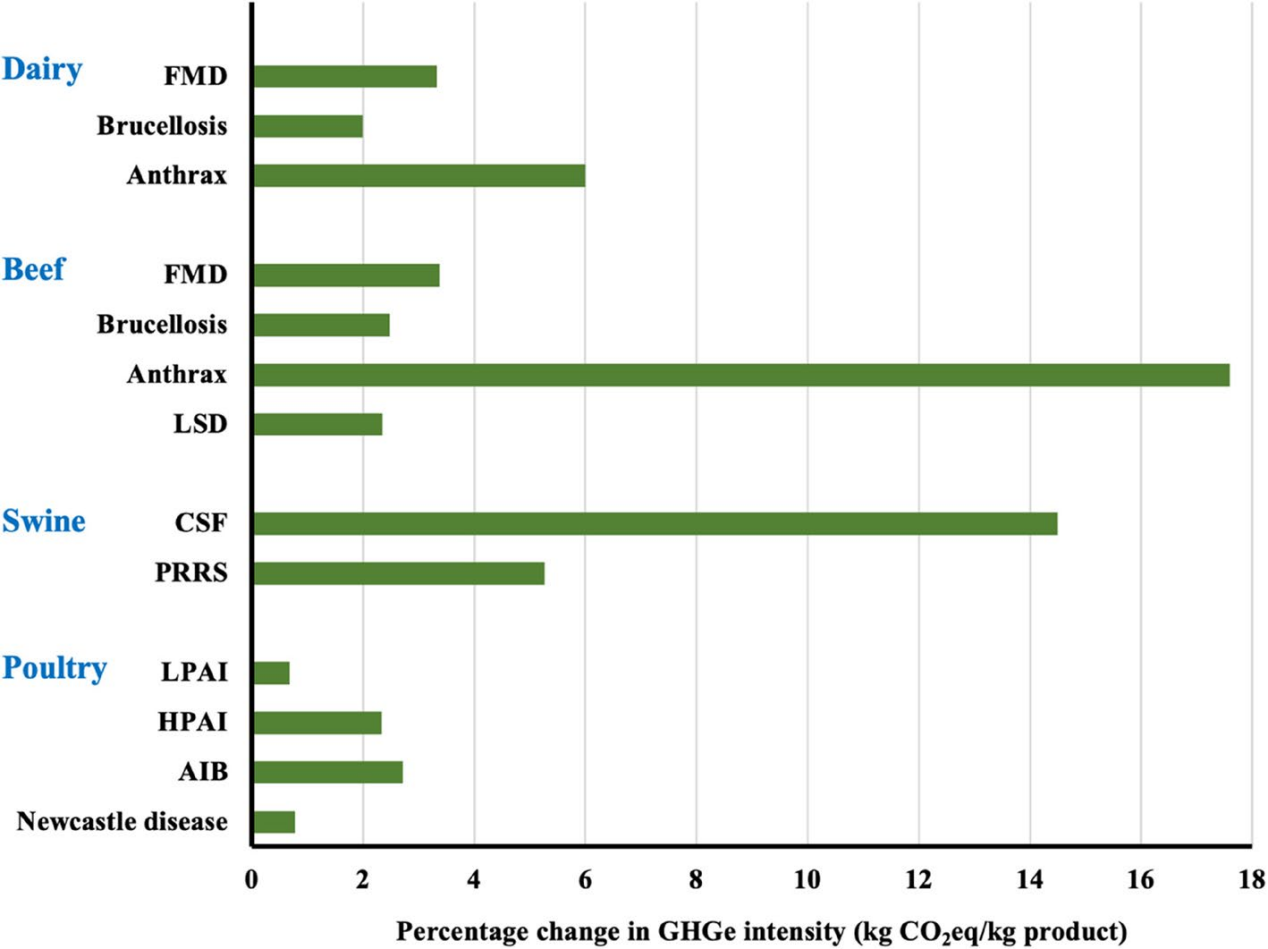
Effects of diseases at a common prevalence (15%) on the % change in GHGe intensity (kg CO_2_eq per kg product) from farm livestock species at the population level

The results of the current study are shown in Tables 8, 9, 10, 11 and 12. Comparing the impacts of different diseases on GHGe per kg of product (milk or meat) across livestock species (Fig. 2) demonstrates that, not unexpectedly, there was a positive association between the extent to which a disease reduced MMY, and the relative increase in GHGe. Diseases that considerably increased mortality (anthrax, CSF, PRRS, HPAI, AIB) had the greatest impact (up to a maximum of 17.6% increase for anthrax in extensive beef cattle) because they required a greater number of livestock in the population to maintain output. By contrast, diseases that had a lesser impact on mortality or produced transient reductions in yield or growth (FMD, brucellosis, LSD, LPAI, Newcastle disease) had a proportionally lower impact on GHGe, ranging from a 0.7% increase (LPAI in backyard poultry) to a 3.5% increase (FMD in extensive beef cattle).

**Table 8:** Impacts of livestock diseases at varying prevalence on GHGe from intensive dairy production at the population (50,000 head) and herd level

Performance parameter	Baseline	High	Low	200-cow herd
**Foot and mouth disease**				
Disease prevalence in population (%)	0	45	5	100
Total annual milk production (metric tonnes FPCM^1^)	101,699	91,041	100,515	690
Change compared to baseline population (%)	-	-10.5	-1.16	-76.7
Total annual calves destined for beef production (head)	12,867	10,549	12,610	66.7
Change compared to baseline population (%)	-	-18.0	-2.00	-33.4
GHGe per kg FPCM^1^ (CO_2_eq)	1.50	1.65	1.52	1.83
Change compared to baseline population (%)	-	10.0	1.11	22.2
Total annual cattle population GHGe (metric tonnes CO_2_eq)	152,549	150,200	152,446	1265
Change in annual cattle population GHGe if milk production maintained (tonnes CO_2_eq)	-	17,585	1796	384
**Brucellosis**				
Disease prevalence in population (%)	0	50	10	40
Total annual milk production (metric tonnes FPCM)	101,699	94,000	100,160	846
Change compared to baseline population (%)	-	-7.57	-1.51	-6.00
Total annual calves destined for beef production (head)	12,867	9751	12,244	91.8
Change compared to baseline population (%)	-	-24.2	-4.84	-8.28
GHGe per kg FPCM^1^ (CO_2_eq)	1.50	1.59	1.52	1.57
Change compared to baseline population (%)	-	5.71	1.14	4.56
Total annual cattle population GHGe (metric tonnes CO_2e_eq)	152,549	149,045	151,954	1327
Change in annual cattle population GHGe if milk production maintained (tonnes CO_2_eq)	-	12,208	2336	84.7
**Anthrax**				
Disease prevalence in population (%)	0	3	0.3	15
Total annual milk production (metric tonnes FPCM^a^)	101,699	100,479	101,577	846
Change compared to baseline population (%)	-	-1.20	-0.12	-6.00
Total annual calves destined for beef production (head)	12,867	12,481	12,829	66.8
Change compared to baseline population (%)	-	-3.00	-0.30	-33.3
GHGe per kg FPCM^1^ (CO_2_eq)	1.50	1.52	1.50	1.59
Change compared to baseline population (%)	-	1.16	0.12	5.87
Total annual cattle population GHGe (metric tonnes CO_2_eq)	152,549	152,469	152,543	1343
Change in annual cattle population GHGe if milk production maintained (tonnes CO_2_eq)	-	1852	183	85.7

**Table 9:** Impacts of livestock diseases at varying prevalence on GHGe from extensive beef production at the population (50,000 head) and herd level

Performance parameter	Baseline	High	Low	200-cow herd
**Foot and mouth disease**				
Disease prevalence in population (%)	0	45	5	100
Total annual beef CW production (metric tonnes)	1619	1392	1594	17.2
Change compared to baseline population (%)	-	-14.0	-1.56	-31.1
Total annual finished cattle produced (head)	4532	3780	4448	44.1
Change compared to baseline population (%)	-	-16.6	-1.84	-36.9
GHGe per kg beef CW (CO_2_eq)	73.0	81.2	73.8	95.7
Change compared to baseline population (%)	-	11.2	1.09	31.1
Total annual cattle population GHGe (metric tonnes CO_2_eq)	118,206	113,034	117,631	1647
Change in annual cattle population GHGe if beef production maintained (tonnes CO_2_eq)	-	18,420	1860	745
**Brucellosis**				
Disease prevalence in population (%)	0	50	10	40
Total annual beef CW production (metric tonnes)	1619	1413	1578	22.4
Change compared to baseline population (%)	-	-12.7	-2.54	-10.2
Total annual finished cattle produced (head)	4532	3800	4386	60.9
Change compared to baseline population (%)	-	-16.2	-3.23	-12.9
GHGe per kg beef CW (CO_2_eq)	73.0	79.7	74.2	78.2
Change compared to baseline population (%)	-	9.13	1.64	7.10
Total annual cattle population GHGe (metric tonnes CO_2_eq)	118,206	112,595	117,084	1755
Change in annual cattle population GHGe if beef production maintained (tonnes CO_2_eq)	-	16,400	3055	199
**Anthrax**				
Disease prevalence in population (%)	0	3	0.3	15
Total annual beef CW production (metric tonnes)	1619	1571	1614	16.8
Change compared to baseline population (%)	-	-3.00	-0.30	-32.8
Total annual finished cattle produced (head)	4532	4396	4518	29.5
Change compared to baseline population (%)	-	-3.00	-0.30	-57.89
GHGe per kg beef CW (CO_2_eq)	73.0	75.3	73.2	79.4
Change compared to baseline population (%)	-	3.09	0.30	8.82
Total annual cattle population GHGe (metric tonnes CO_2_eq)	118,206	118,206	118,206	1333
Change in annual cattle population GHGe if beef production maintained (tonnes CO_2_eq)	-	3656	356	652
**Lumpy skin disease**				
Disease prevalence in population (%)	0	8.0	2.5	30
Total annual beef CW production (metric tonnes)	1619	1587	1609	23.1
Change compared to baseline population (%)	-	-2.01	-0.63	-7.54
Total annual finished cattle produced (head)	4532	4423	4498	63.6
Change compared to baseline population (%)	-	-2.40	-0.75	-10.0
GHGe per kg beef CW (CO_2_eq)	73.0	73.9	73.3	76.6
Change compared to baseline population (%)	-	1.22	0.38	4.87
Total annual cattle population GHGe (metric tonnes CO_2_eq)	118,206	117,249	117,907	1769
Change in annual cattle population GHGe if beef production maintained (tonnes CO_2_eq)	-	2404	745	144

**Table 10:** Impacts of livestock diseases at varying prevalence on GHGe from swine production at the population (100,000 sows) and herd level

Performance parameter	Baseline	High	Low	1000-sow herd
**Classical swine fever**				
Disease prevalence in population (%)	0	20	5	90
Total annual pork CW production (metric tonnes)	161,808	139,093	156,129	596
Change compared to baseline population (%)	-	-14.0	-3.5	-63.2
Total annual pigs sold (‘000 head)	1935	1663	1867	7.126
Change compared to baseline population (%)	-	-14.0	-3.5	-63.2
GHGe per kg pigmeat CW (CO_2_eq)	6.07	7.27	6.36	15.0
Change compared to baseline population (%)	-	19.7	4.7	147.1
Total annual pig population GHGe (metric tonnes CO_2_eq)	982,614	929,138	971,831	5247
Change in annual pig population GHGe if pigmeat production maintained (tonnes CO_2_eq)	-	160,470	35,740	16,855
**Porcine reproductive and respiratory syndrome**				
Disease prevalence in population (%)	0	60	10	70
Total annual pork CW production (metric tonnes)	161,808	113,173	153,248	1057
Change compared to baseline population (%)	-	-30.1	-5.3	-34.7
Total annual pigs sold (head)	1,935,000	1,353,399	1,832,644	12,639
Change compared to baseline population (%)	-	-30.1	-5.3	-34.7
GHGe per kg pigmeat CW (CO_2_eq)	6.07	8.19	6.35	8.70
Change compared to baseline population (%)	-	34.9	4.5	43.3
Total annual pig population GHGe (metric tonnes CO_2_eq)	982,614	920,871	971,253	9121
Change in annual pig population GHGe if pigmeat production maintained (tonnes CO_2_eq)	-	422,262	54,881	5217

**Table 11:** Impacts of livestock diseases at varying prevalence on GHGe from backyard poultry production at the population (100,000 hens) and flock level

Performance parameters	Baseline	High	Low	10-hen flock
**Low pathogenicity avian influenza**				
Disease prevalence in population (%)	0	50	10	90
Total annual poultry CW production (metric tonnes)	1146	1104	1138	0.107
Change compared to baseline population (%)	-	-3.7	-0.7	-6.7
Total annual chickens sold (‘000 head)	1819	1752	1806	0.170
Change compared to baseline population (%)	-	-3.7	-0.7	-6.7
GHGe per kg poultry meat CW (CO_2_eq)	10.31	10.56	10.36	10.79
Change compared to baseline population (%)	-	0.25	0.05	0.48
Total annual poultry population GHGe (metric tonnes CO_2_eq)	11,821	11,658	11,786	1.154
Change in annual population GHGe if poultry production maintained (tonnes CO_2_eq)	-	454	88.1	.084
**High pathogenicity avian influenza**				
Disease prevalence in population (%)	0	70	30	100
Total annual poultry CW production (metric tonnes)	1146	587	886	0.040
Change compared to baseline population (%)	-	-48.8	-22.7	-65.1
Total annual chickens sold (‘000 head)	1819	932	1406	0.063
Change compared to baseline population (%)	-	-48.8	-22.7	-65.1
GHGe per kg poultry meat CW (CO_2_eq)	10.31	12.24	10.87	14.19
Change compared to baseline population (%)	-	18.7	5.4	37.6
Total annual poultry population GHGe (metric tonnes CO_2_eq)	11,821	6301	9204	0.452
Change in annual population GHGe if poultry production maintained (tonnes CO_2_eq)	-	11,255	3475	2208
**Avian infectious bronchitis**				
Disease prevalence in population (%)	0	75	20	95
Total annual poultry CW production (metric tonnes)	1146	921	1085	0.086
Change compared to baseline population (%)	-	-19.6	-5.4	-24.7
Total annual chickens sold (‘000 head)	1819	1462	1722	0.137
Change compared to baseline population (%)	-	-19.6	-5.4	-24.7
GHGe per kg poultry meat CW (CO_2_eq)	10.31	12.05	10.69	12.71
Change compared to baseline population (%)	-	16.9	3.6	23.3
Total annual poultry population GHGe (metric tonnes CO_2_eq)	11,821	10,881	11,532	1.069
Change in annual population GHGe if poultry production maintained (tonnes CO_2_eq)	-	2890	670	0.388
**Newcastle disease**				
Disease prevalence in population (%)	0	80	25	95
Total annual poultry CW production (metric tonnes)	1146	982	1095	0.095
Change compared to baseline population (%)	-	-14.3	-4.5	-17.0
Total annual chickens sold (‘000 head)	1819	1559	1738	0.151
Change compared to baseline population (%)	-	-14.3	-4.5	-17.0
GHGe per kg poultry meat CW (CO_2_eq)	10.31	10.97	10.45	11.18
Change compared to baseline population (%)	-	6.4	1.3	8.4
Total annual poultry population GHGe (metric tonnes CO_2_eq)	11,821	10,278	11,285	1.003
Change in annual population GHGe if poultry production maintained (tonnes CO_2_eq)	-	1971	556	0.242

**Table 12:** Effect of changing disease prevalence via effective control measures (e.g. vaccination) on GHGe per kg of milk or meat

	**Prevalence**		**GHGe, CO**_**2**_**eq per kg of milk or meat**^**a**^		
**Livestock system**	**Initial**	**After control**	**Initial**	**After control**	**Change**^**b**^** (%)**
**Dairy**					
Foot and mouth disease	45	5.0	1.65	1.52	-7.88
Brucellosis	50	10	1.59	1.52	-4.40
Anthrax	3.0	0.3	1.52	1.50	-1.32
**Beef**					
Foot and mouth disease	45	5.0	81.2	73.8	-9.11
Brucellosis	50	10	79.7	74.2	-6.90
Anthrax	3.0	0.3	75.3	73.2	-2.79
Lumpy skin disease	8.0	2.5	73.9	73.3	-0.81
**Swine**					
Classical swine fever	20	5.0	7.27	6.36	-12.5
Porcine reproductive and respiratory syndrome	60	10	8.19	6.35	-22.5
**Poultry**					
Low pathogenicity avian influenza	50	10	10.56	10.36	-1.89
High pathogenicity avian influenza	70	30	12.24	10.87	-11.2
Avian infectious bronchitis	75	20	12.05	10.69	-11.3
Newcastle disease	80	25	10.97	10.45	-4.74

^a^ Kg of fat and protein corrected milk used as the denominator for dairy cattle, kg of carcass weight meat for beef, swine and poultry

^b^ Change refers to the % change in GHGe per kg of milk or meat after disease control

As disease prevalence increased, the impact of disease on MMY and GHGe also increased in all species and systems investigated (Tables 8, 9, 10, 11 and 12). for example, compared to the baseline, FMD resulted in an 1.11% increase in the GHGe per kg of milk at a 5% prevalence, compared to a 10.0% increase in GHGe per kg milk at a 45% prevalence (Table 8). Similarly, the impact of PRRS on GHGe from swine production varied from a 4.5% increase in GHGe per kg meat at a 10% prevalence, to a 34.9% increase at a 60% prevalence (Table 10).

## Discussion

Sustainable food production comprises a balance between economic viability, environmental responsibility and social acceptability [48]. Multiple studies have evaluated the impacts of livestock diseases on economic viability [31, 32, 39, 49, 50], although such studies date quickly and are often region-specific. However, more research is urgently needed to fill the current knowledge gap relating to the impact of livestock disease and its prevention or control on the second pillar of sustainability – environmental responsibility – and on all three pillars in combination.

In accordance with previous work reporting the effects of livestock health on environmental impacts, GHGe were increased when maintaining milk or meat output from systems impacted by livestock disease [51–54]. This increase is primarily due to the inverse of the dilution of maintenance effect, whereby a reduction in livestock MMY necessitates a greater number of livestock and/or a longer time period being required to produce a set amount of LDF [55]. Diseases that considerably increased mortality (e.g. anthrax) had greater impacts than those that affected growth or reproduction, and diseases that increase mature animal mortality have a greater impact than those associated with neonatal or growing animal deaths because of the resources already invested in raising the animal to that point. Similar effects of disease on system efficiency have been previously reported, whereby Johnes disease was cited as increasing GHGe by 25% per kg of milk and 40% per kg of beef due to long-term effects on feed efficiency and growth before premature culling, whereas diarrhoea in dairy calves, with short-lived effects on intake and growth in heifer replacements increased GHGe by less than 1% per kg of milk [56].

Some of the data in this study were normalised to a standard prevalence to allow a theoretical comparison across species and diseases (Fig. 2), although under real-life conditions, effects of disease are confounded by prevalence and these impacts are significant data gaps in the literature. Within a specific species, the MMY impacts increase with disease prevalence – the GHGe effects of a specific disease at the population or herd level therefore derive from a function of MMY effects and prevalence. For example, anthrax is inevitably fatal, causing significant MMY losses, yet the majority of anthrax cases are caused by ingestion of spores from soil or pasture, transmission between animals is rare and prevalence (at the population level) is low. By contrast, brucellosis has a far lesser impact on cattle MMY, yet is highly contagious between infected cattle and can spread quickly and easily within a herd or population (Table 6). Despite these differences, the two diseases conferred almost identical increases in total GHGe from a 200-cow dairy herd within the current study, but had very different impacts at the population level (Table 8). It is therefore essential to consider the relative merits of mitigating impacts of widespread but relatively low GHGe impact diseases, or those that have considerable impacts at the localised level, yet only affect a relatively small proportion of herds or flocks.

The global effects of absolute increases in GHGe conferred by disease are further complicated by the size of livestock populations. Worldwide, considerably more head of poultry and swine exist than ruminant animals, yet the bodyweight of an average dairy cow is ~ 500 × than of a hen. Consequently, cattle account for 54.8% of global livestock mass, compared to 18.4% for swine and 3.4% for chickens (Author’s calculation derived from supplementary data from [57]). The potential impacts of diseases within cattle production on both environmental impacts and LDF losses are further exacerbated by the greater amount of time required from birth to slaughter (and therefore greater total days-at-risk of disease) compared to growing swine or growing chickens, and the average GHGe per kg of milk or meat [11, 12, 58]. In developed regions, commercial swine and poultry operations tend to be more integrated than their ruminant counterparts and have a greater adoption of preventative veterinary strategies. Considerable potential gains may therefore exist from improved disease control within beef production systems in South Asia, Latin America and sub-Saharan Africa, cited as having the highest regional GHGe per kg of CW [12]. This is underlined by the results shown in Table 9 – if LSD was controlled in a 200-cow beef herd, the GHGe intensity would be reduced from 76.6 kg CO_2_eq per kg CW to 73.0 kg CO_2_eq per kg CW, with further gains made by improved hide quality.

Changes in GHGe associated with attempting to maintain MMY from livestock systems challenged by disease are difficult to conceptualise in isolation but for greater context can be compared to the equivalent annual emissions from passenger vehicles (cars) based on equivalent annual exhaust emissions from United Kingdom passenger vehicles with emissions of 0.152 kg CO_2_ per km driven and 11,909 km driven per year [59, 60]. For example, at low population prevalence levels, the effect of disease on GHGe was relatively minor, ranging from an increase equivalent to annual emissions from 49 vehicles for LPAI in poultry at 10% prevalence (Table 11), to 30,358 car-equivalents of GHGe for PRRS in swine at 10% prevalence (Table 10). However, at high prevalence levels, the totals increased to 251 car-equivalents for LPAI in poultry at 50% prevalence to 233,578 car-equivalents for PRRS in swine at 60% prevalence. At the farm level (herd or flock) this can give an insight into the practical impacts of disease. For example, brucellosis at 40% prevalence in a 200-cow beef herd increased GHGe (Table 9) by the equivalent of the annual emissions from 110 cars. By contrast, CSF at 90% prevalence in a 1,000-sow herd (Table 10) increased GHGe by the equivalent of the annual emissions from 9,324 cars; whereas Newcastle disease at 95% prevalence in a 10-bird poultry flock (Table 11) increased GHGe by the equivalent of the annual emissions from 0.13 cars. It is acknowledged that vehicle emissions vary considerably between countries and change over time, yet it is important to understand the context of changes in livestock GHGe compared to other GHG sources.

Livestock producers worldwide must improve health and welfare through improved disease surveillance and reporting, optimized medicines use, implementation of preventative herd or flock health plans and adoption of tools and technologies that improve and facilitate these goals. This requires a concerted effort to improve collaboration and communication between all livestock production stakeholders, from the producer, veterinary surgeon, nutritionist and geneticist through the processor and retailer to the policymaker and consumer. The new OIE-WAHIS portal [15] was developed with the intent of facilitating disease reporting, increasing and easing data reporting and therefore improving global data quality. If successful, this will bridge the data gap relating to livestock disease, productivity and GHGe; however, it is too early to judge whether these aims have been met. The global threat presented by antimicrobial resistance (AMR) to human, animal and ecosystem health and the consequent need to reduce, replace and refine antimicrobial use offers a clear rationale for implementing preventative health programmes for livestock across the globe, yet the advantages of these programmes must be clearly communicated to encourage producer uptake [61]. The results of the current study add to the body of knowledge and information that may be used in this drive to increase vaccine use. The effectiveness and adoption of vaccines will never reach 100% across a real-life livestock population. Nevertheless, the potential reductions in milk and meat GHGe intensity (kg CO_2_eq/kg product) and improved food security that can be conferred by disease prevention should be of considerable interest to processors, retailers and policymakers. For example, reducing the prevalence of PRRS in swine populations from 60 to 10% would reduce GHGe intensity by 22.5%; whereas reducing FMD in beef cattle from 45 to 5% prevalence, or AIB in poultry from 75 to 20% prevalence would reduce GHGe intensities by 9.11% and 11.3% respectively (Table 12). This is not intended to suggest that PRRS is the most important disease warranting control, or indeed that swine are more deserving of disease control measures than other animals. Indeed, there is need for a comprehensive global disease analysis that allows diseases to be compared on the basis of sustainability impacts. Although vaccines are available for all the diseases investigated within the current study, they are not available or adopted within every region or system worldwide due to economic, political, infrastructure or veterinary constraints. Outreach and extension programmes that disseminate information and enhance producer knowledge relating to the sustainability benefits of vaccines on economic viability and environmental responsibility must be developed—participatory approaches that enhance peer-to-peer learning were shown to improve medicines use on dairy farms – given the value of farmer discussions in changing behaviour, the same approach may be adopted worldwide [62]. Lessons can and should be learned from the global vaccine response to the COVID-19 pandemic in terms of the ability to rapidly develop and disseminate effective preventative healthcare, although the limitations of research funding and inequalities between high- and low-income countries must be acknowledged and addressed [63].

As shown by the poultry-related results of this study, controlling disease in backyard flocks would significantly improve output and therefore livelihoods of many smallholder and subsistence livestock producers across the globe. Globally, livestock ownership provides myriad benefits beyond income, including improved nutrition, health, cultural status, education, female emancipation and asset diversification [64, 65]. The potential sustainability impacts of improving animal health therefore extend beyond GHGe and economic viability of the individual farm to whole system and community sustainability, particularly in areas containing a high proportion of smallholder farmers, who are inherently vulnerable to risk and who would benefit considerably from vaccine adoption [66].

The importance of controlling animal disease as a strategy to improve food security across all systems must be included in sustainability discussions. Although the statistic is now dated, over 20% of global animal protein has been cited as lost because of livestock disease, and these are primarily diseases for which treatments already exist, yet are not adopted [67]. At the farm-level, every growing or finishing animal (i.e. those destined for slaughter rather than breeding) that dies results in a loss of potential beef, pigmeat or poultry meat equal to its CW yield. However, the loss of a breeding cow, sow or hen also results in a greater potential food loss in the opportunity cost of milk or offspring that, in the absence of disease, would have been produced, and the need to divert female offspring into replacements rather than meat. Assuming that breeding livestock die, on average, halfway through their production cycle, this loss plus the time required to produce a replacement animal will be equal to total losses of 8,357 kg milk (dairy cattle), 344.7 kg CW of prime beef, 1,622 kg CW pigmeat or 5.7 kg CW poultry meat. These numbers need to be put into context to be meaningful, however, so, for example, the average poultry meat consumption in Malaysia is 53.1 kg boneless meat per person [68], therefore the potential CW gains from controlling HPAI in an area containing a total of 100,000 hens within backyard flocks (plus associated growing chickens) at a 70% prevalence (Table 11) would supply 7,370 people in Malaysia with their annual poultry meat demand. Similarly, if classical swine fever was eliminated from pig production, the improved output from a 1,000-sow herd at a 90% prevalence (Table 10) would supply 32,868 people in China with their annual pigmeat demand, based on 31.1 kg CW consumed annually per capita [68]. The recent outbreak of African swine fever, with swine losses cited at between 150–200 million animals [50] would therefore, at a minimum (assuming only growing animals died) represent food losses equivalent to the annual consumption of 403–538 million people in China (up to 38% of the population), yet, as previously discussed, this is an underestimate as it does not include pigmeat opportunity cost.

This study reveals the impact of disease on GHGe per kg of milk or meat produced, and therefore the potential gains through either through effective disease control (reduced prevalence, Table 12) or elimination (Additional file 1). This study deliberately did not aim to definitively quantify the impacts of disease or it’s control on specific populations or regions. Without current, precise information on global disease prevalence and performance impacts it is virtually impossible to accurately predict the regional or global GHG implications of, for example, vaccine use, as effects will vary according to disease identification and prevalence, individual vaccine efficacy and effectiveness, and adoption and use by livestock producers. These factors are further complicated by variation in GHGe between regions, systems and livestock species, plus the emissions conferred by vaccine manufacture, transport and use. At present, estimates of disease prevalence are often missing, dated, or considerably underestimated [69] and effects on key performance indicators (e.g. mortality, yields, growth rate and carcass quality) exhibit such variation between systems, regions and climates that ascribing a specific mortality or production loss to a disease will inevitably under or overestimate the impacts – an acknowledged limitation of this study. This could, in future studies, be mitigated by expanding the scope to include sensitivity analyses to examine a range of prevalence and performance impacts per disease, in addition to widening the number of systems and regions examined. Nevertheless, within the confines of the current study, results indicate that considerable gains may be made by vaccinating livestock such that disease prevalence is reduced and therefore performance maintained. These gains include potential reductions in the GHGe intensity per kg of milk ranging from 1.32% (anthrax) to 7.88% (FMD); per kg of beef from 0.81% (LSD) to 9.11% (FMD); per kg of pigmeat from 12.5% (CSF) to 22.5% (PRRS); and per kg of poultry meat from 1.89% (LPAI) to 11.3% (AIB), as shown in Table 12. Further gains would be made by completely eliminating disease, with reductions in GHG per kg of meat ranging from 0.27% (low prevalence anthrax in beef cattle) up to 25.9% (high prevalence PRRS in swine).

Modelling studies confer both opportunities and limitations in terms of the breadth, depth and accuracy of the results—the current study was therefore constrained by a number of factors. An urgent need exists to collect, update and benchmark data relating to global disease incidence and losses – although the trends in relative importance of animal diseases are unlikely to have changed substantially since the publication used in this study [16], relying on data that is 10 + years old as a basis for choosing diseases to investigate is less than ideal. It is hoped that this may be rectified in future by the recent multi-year initiative to evaluate the global burdens of animal diseases [70]. Although the current results may be used as a foundation or guide for discussion, they should not be taken as a definitively accurate assessment. Few diseases occur in isolation, therefore additional modelling should be undertaken to account for interactions between diseases within livestock populations and the impacts of concomitant and secondary disease on MMY losses. Indeed, as many diseases co-exist and interact on-farm, it is difficult to quantify the effects of a single disease on economic or environmental impacts [8, 71]. The methodology by which this is assessed is crucial however: a recent study examined three options for assessing economic burdens of co-existing endemic disease in UK dairy cattle, finding that the costs of aggregated diseases were less than would have been predicted from non-aggregated data, yet disease rankings varied considerably according to the methodology used [72].

Within the current study, animals were assumed to die halfway through their production, breeding or growth cycle (unless aborted or stillborn), therefore account for the embedded GHGe invested in producing animals that then die and do not enter the food chain. However, although the total LDF losses might not change, the GHGe associated with mortality of a 2-day-old chicken would differ greatly from a beef steer being lost at 30 mo of age, therefore the timing of morbidity and mortality also warrants further investigation. The consequential implications of disease incidence and co-products would also reveal a more complete picture of the impacts of livestock health than can be gained from examining different livestock species or systems in isolation. Examples would include the effects on total beef production and GHGe resulting from disease outbreaks in dairy cattle that result in fewer male calves being produced, or a shift towards greater poultry production and consumption in the event of a disease outbreak in swine. Finally, given the importance of climate change in public and policy debate, and the MMY impact of the diseases investigated within the current study, it is unfortunate that there appear to be no papers in the literature have investigated disease effects upon GHGe, although a body of literature exists examining the impacts of climate change on future disease incidence and epidemiology. As livestock producers become more aware of the importance of improving health and reducing GHGe, this knowledge gap will urgently need to be filled. Ideally, this would be achieved through implementation of an accurate and continuous global disease surveillance monitoring and reporting system, coupled with improved on-farm GHG assessment tools and metrics.

## Conclusion

Livestock health is an integral component of sustainability via the impacts of morbidity and mortality on MMY and therefore on both LDF output and GHGe. This study shows that reducing the prevalence or eliminating diseases that have negative impacts on milk and meat output should reduce the GHGe intensity (kg CO_2_eq/kg product) of LDF production, although the magnitude of specific disease effects varies according to the degree of output losses, disease prevalence and the characteristics of the baseline population. Controlling or eliminating diseases of global importance may have considerable benefits in terms of improving food security and mitigating the impacts of livestock production on the environment. Implementing a culture of continuous improvement, including data collection, recording and benchmarking disease impacts so that their effects can be effectively quantified and communicated to stakeholders throughout the food system will allow evidence-based decisions to be made at the farm, processor, retailer and policy level. Given concerns over AMR, improving the adoption and application of vaccines to control diseases within global livestock production offers significant opportunities to enhance both livestock system sustainability and One Health.

### Supplementary Information

**Additional file 1.**

## Data Availability

All the principal data generated and analysed within the study are included in this article within the main tables, but on reasonable request, the full dataset and further details of the models are available from the corresponding author.
