# Supplementary material for: The impact of controlling diseases of significant global importance on greenhouse gas emissions from livestock production
Source: One Health Outlook. 2023 Dec 8;5:17. doi: 10.1186/s42522-023-00089-y (PMC10704630; doi:10.1186/s42522-023-00089-y)
Supplement: Supplementary file 1 — Additional file 1. [file 42522_2023_89_MOESM1_ESM.docx]

**Appendix 1. Effect of moving from a high or a low prevalence of livestock disease to disease elimination^a^ via effective control measures (e.g. vaccination) upon GHGe per kg of milk or meat^b^**

|  | **High prevalence** | | | **Low prevalence** | | |
| --- | --- | --- | --- | --- | --- | --- |
| **Livestock system** | **Prevalence (%)** | **GHGe, CO_2_eq per kg of milk or meat^b^** | **Percentage change (%) if eliminated** | **Prevalence (%)** | **GHGe, CO_2_eq per kg of milk or meat^b^** | **Percentage change (%) if eliminated** |
| **Dairy** |  |  |  |  |  |  |
| Foot and mouth disease | 45 | 1.65 | -9.09 | 5.0 | 1.52 | -1.32 |
| Brucellosis | 50 | 1.59 | -5.66 | 10 | 1.52 | -1.32 |
| Anthrax | 3.0 | 1.52 | -1.32 | 0.3 | 1.50 | -0.28 |
| **Beef** |  |  |  |  |  |  |
| Foot and mouth disease | 45 | 81.2 | -10.1 | 5.0 | 73.8 | -1.08 |
| Brucellosis | 50 | 79.7 | -8.41 | 10 | 74.2 | -1.62 |
| Anthrax | 3.0 | 75.3 | -3.05 | 0.3 | 73.2 | -0.27 |
| Lumpy skin disease | 8.0 | 73.9 | -1.22 | 2.5 | 73.3 | -0.41 |
| **Swine** |  |  |  |  |  |  |
| Classical swine fever | 20 | 7.27 | -16.5 | 5.0 | 6.36 | -4.56 |
| Porcine reproductive and respiratory syndrome | 60 | 8.19 | -25.9 | 10 | 6.35 | -4.41 |
| **Poultry** |  |  |  |  |  |  |
| Low pathogenicity avian influenza | 50 | 10.56 | -2.37 | 10 | 10.36 | -0.48 |
| High pathogenicity avian influenza | 70 | 12.24 | -15.8 | 30 | 10.87 | -5.15 |
| Avian infectious bronchitis | 75 | 12.05 | -14.44 | 20 | 10.69 | -3.55 |
| Newcastle disease | 80 | 10.97 | -6.02 | 25 | 10.45 | -1.34 |

^a^ Elimination in this instance is equal to the baseline values quoted in tables 9-12

^b^ Kg of milk used as the denominator for dairy cattle, kg of meat for beef, swine and poultry
